# The development of insecticide-treated durable wall lining for malaria control: insights from rural and urban populations in Angola and Nigeria

**DOI:** 10.1186/1475-2875-11-332

**Published:** 2012-09-18

**Authors:** Louisa A Messenger, Nathan P Miller, Adedapo O Adeogun, Taiwo Samson Awolola, Mark Rowland

**Affiliations:** 1 Faculty of Infectious Tropical Diseases, London School of Hygiene and Tropical Medicine, London, UK; 2 The MENTOR Initiative, Huambo, Angola; 3 Molecular Entomology and Vector Control Research Laboratory, Nigerian Institute of Medical Research, Lagos, Nigeria

**Keywords:** Durable wall lining, ZeroVector®, Insecticide-treated plastic sheeting, Malaria control, Acceptability

## Abstract

**Background:**

Durable lining (DL) is a deltamethrin-impregnated polyethylene material, which is designed to cover domestic walls that would normally be sprayed with residual insecticide. The operational success of DL as a long-lasting insecticidal substrate will be dependent on a high level of user acceptability as households must maintain correctly installed linings on their walls for several years. Preliminary trials were undertaken to identify a material to develop into a marketable wall lining and to assess its level of acceptability among rural and urban populations.

**Methods:**

In Angola (n=60), prototype DL and insecticide-treated plastic sheeting (ITPS) were installed on urban house walls and ceilings, respectively, and acceptability was compared to indoor residual spraying (IRS) (n=20) using a knowledge, attitude and practice (KAP) questionnaire. In Nigeria (n=178), three materials (prototype DL, ITPS and insecticide-treated wall netting) were distributed among rural and urban households. User opinions were gathered from focus group discussions, in-depth interviews and KAP questionnaires.

**Results:**

In Angola, after two weeks, the majority of participants (98%) expressed satisfaction with the products and identified the killing of insects as the materials’ principal benefits (73%). After one year, despite a loss of almost 50% of households to refugee repatriation, all 32 remaining households still asserted that they had liked the DL/ITPS in their homes and given the choice of intervention preferred DL/ITPS to IRS (94%) or insecticide-treated nets (78%). In Nigeria, a dichotomy between rural and urban respondents emerged. Rural participants favoured wall adornments and accepted wall linings because of their perceived decorative value and entomological efficacy. By contrast, urban households preferred minimal wall decoration and rejected the materials based upon objections to their aesthetics and installation feasibility.

**Conclusions:**

The high level of acceptability among rural inhabitants in Nigeria identifies these communities as the ideal target consumer group for durable wall linings. The poorer compliance among urban participants suggests that wall linings would not be readily adopted or sustained in these regions. If DL is as well received by other rural populations it could overcome some of the logistical constraints associated with spray campaigns and has the potential to become a long-lasting alternative to IRS in malaria endemic areas.

## Background

Successful malaria vector control is dependent on sustained user cooperation, logistical feasibility and the existence of appropriate delivery systems. The two principal methods of malaria vector control, advocated by the World Health Organization (WHO), are indoor residual spraying (IRS) and long-lasting insecticidal nets (LLINs) [1]. Choice of vector control is largely governed by epidemiological and operational circumstances, as both LLINs and IRS have demonstrated equivalent levels of efficacy [2]. IRS is appropriate to control unstable or epidemic malaria with the advantage of achieving rapid vector population suppression in areas of high disease risk [3]. However, the logistics and infrastructure required to mount and execute repeated spray campaigns are impractical in rural areas afflicted with seasonal malaria [4]. By contrast, LLINs are more cost-effective [5] and less technically demanding to implement, allowing targeted distribution to the most at-risk individuals, such as pregnant women and children, or to entire communities via universal coverage campaigns. Drawbacks associated with adherence and retention of LLINs include personal confinement and discomfort when humidity and indoor temperatures are high [6].

The search for novel, acceptable and affordable methods of vector control for marginalized populations and complex emergencies has thus far yielded a number of inventive solutions [7-11]. In particular, suppression of malaria vector populations achieved using insecticide-treated tarpaulins [12,13] and tents [14] in refugee camps has stimulated an interest in exploiting such ubiquitous materials to create a sustainable community-level substitute to IRS to use during peacetime. Long-lasting durable lining (DL) fixed to walls and/or ceilings indoors could overcome some of the logistical constraints associated with repeated rounds of spraying, whilst retaining the most attractive feature of IRS, the protection of all members of the community.

DL is currently manufactured commercially (ZeroVector®, Vestergaard Frandsen, Switzerland) as a thin sheet of woven high-density polyethylene (HDPE) shade cloth with deltamethrin (4.4 g/kg ± 15% *a.i.*) incorporated during production; it is designed to cover interior wall surfaces and remain efficacious for three to four years. The acceptability, durability and bioefficacy of this product have recently been assessed in a multicentre field trial where ZeroVector® DL remained fully efficacious against mosquito vectors, demonstrated minimal loss of insecticide content over 12 months of field use and was unequivocally more popular than IRS and other long-lasting vector control products [15].

The operational success of DL is heavily reliant on its level of household acceptability as occupants must be motivated to maintain correctly installed materials on their house walls for up to four years at a time. In order to sustain user compliance, the DL must be perceived to greatly benefit the household, either through the elimination of vectors or nuisance insects and/or the provision of household decoration. This report describes the initial development of several prototype durable wall lining materials into a desirable and marketable vector control product through a series of pilot field trials in Angola and Nigeria. The aims of these studies were to establish an appropriate target demographic and gain an in-depth understanding of rural and urban communities as consumers in order to design improved products and distribution systems to reach them effectively.

## Methods

### Study sites and household installation

In August 2005 a pilot study was conducted in Angola to assess the levels of interest in DL among urban households in order to identify an appropriate target market for this vector control product. In that year alone, over four million clinical cases of malaria were reported in Angola, resulting in 20,000 deaths and accounting for over 35% and 25% of mortality in children under five and maternal deaths, respectively [16]. Huambo province (12^o^46^′^S; 15^o^44^′^E) is an area in the subtropical highlands of central Angola. Malaria (*Plasmodium falciparum*) is stable and meso-endemic, with transmission peaking during the rainy season from October to April, by *Anopheles gambiae sl.* and *Anopheles funestus*[17-19].

**Figure 1:**
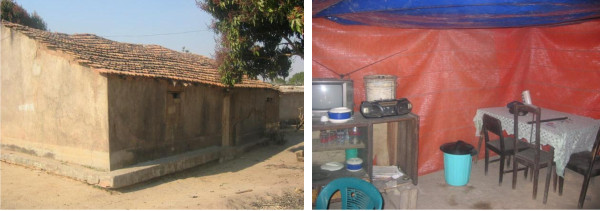
**Cacilhas, Huambo Province, Angola.** Sixty households (left) in an urban area of Huambo Province, Angola, received a polyethylene shade cloth durable lining (DL) and a heavy-weight insecticide-treated plastic sheeting (ITPS) to cover the walls and ceilings of their houses, respectively (right).

Sixty houses in an urban area (Cacilhas), on the outskirts of Huambo province, received a prototype polyethylene shade cloth durable lining (henceforth DL) (80 g/m^2^) and a heavy-weight insecticide-treated plastic sheeting (ITPS) (105 g/m^2^) (supplied by Vestergaard Frandsen, Switzerland) to cover the walls and ceilings of their houses, respectively (Figure 1). Both prototype DL and ITPS were designed based upon LLIN technology; deltamethrin (at 3.15 g/kg ± 25% *a.i.*) was incorporated into the polyethylene polymer during the manufacturing process, allowing the weave matrix to act as a reservoir, which regulates the migration of insecticide to the surface. Three different-coloured DLs (blue, green and orange) were manufactured and distributed randomly among the households. All houses also received blue ITPS. The DL was fixed over the entire wall, including doors, windows and eaves, and the ITPS was used to cover the ceiling and attached to the top of the DL. Both materials were attached to walls and ceilings using a minimum of 12 nails per room. In houses with multiple rooms, DL/ITPS was installed in all rooms. The DL and ITPS were installed in the first few houses by trained staff, while two community members observed. Subsequent households were given the materials and occupants were responsible for the installation with assistance from trained personnel and community members. An additional 20 houses from an adjacent area, which had received IRS on all house walls (lambda-cyhalothrin capsule suspension 100 g/l (ICON® 10 CS; Syngenta) at an application dose of 25 mg/m^2^ using a standard 10 L Hudson X-pert pump), were selected to participate in the study. All houses were small (one to three rooms), built from mud brick adobe with thatched/slate roofing, and of similar structure and construction to rural houses in Huambo.

**Figure 2:**
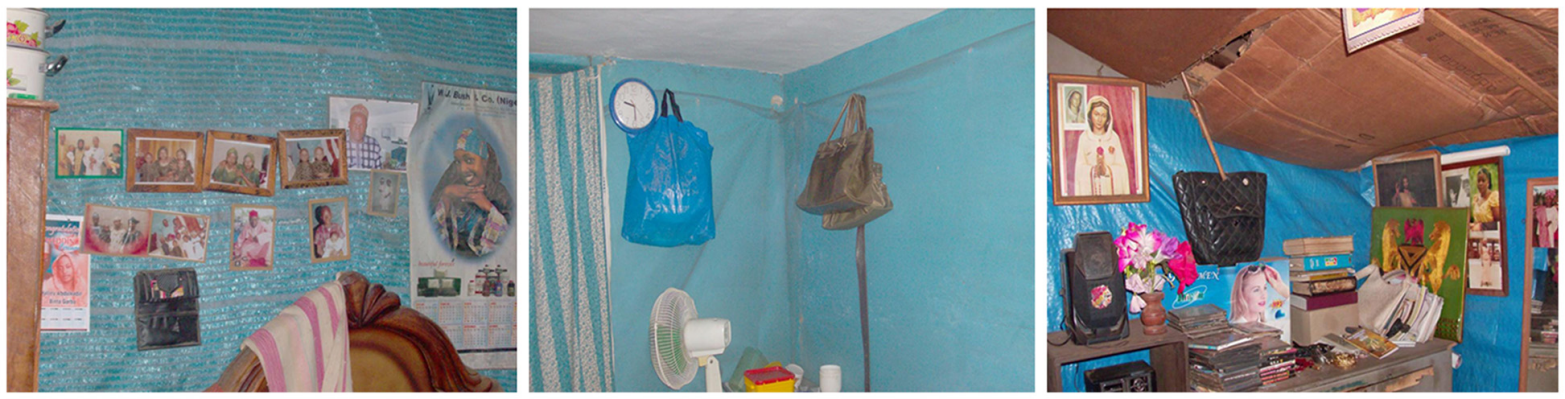
Examples of prototype DL (left), wall netting (middle) and ITPS (right) materials installed in houses in Enugu, Nigeria.

In December 2006 a preliminary trial was undertaken in Nigeria to establish a desirable material to use as a durable wall covering. Nigeria accounts for a quarter of all malaria cases in the WHO Africa Region [20] and over 300,000 individuals, principally pregnant women and children under five, die of this disease annually [21]. Three insecticide-treated prototype lining materials – DL, ITPS (both previously assessed in Angola) and wall netting - were evaluated among rural and urban participants in Lagos, Enugu and Kano (Figure 2). Wall netting was made from PermaNet 2.0 polyester fabric (40 g/m^2^) with deltamethrin incorporated at 1.8 g/kg ± 10% *a.i.* (also supplied by Vestergaard Frandsen, Switzerland).

Lagos (6^o^27^′^11^”^N; 3^o^23^′^45^”^E) is the commercial capital of Nigeria, situated on the south-western Atlantic seaboard, with an estimated population of 17 million [22]. Enugu (6^o^27^′^9.60^”^N; 7^o^30^′^37.20^”^E) is located in the south-east of Nigeria. Both Lagos and Enugu have a tropical savannah climate with rainy seasons from April to October. Kano (12^o^00^’^N; 8^o^31^’^E) is a city in north-central Nigeria that contains the second largest metropolitan population after Lagos. The climate is hot and semi-arid with a rainy season from June to September.

A total of 178 households in Lagos (61), Enugu (60) and Kano (57) were recruited to receive one of the three prototype lining materials. Ninety-one of these were rural houses while the rest were in urban areas. DL was installed in households in rural Lagos (31/61), urban and rural Enugu (10/60 and 15/60, respectively) and urban and rural Kano (9/57 and 15/57, respectively). Wall netting was only installed in urban houses in Lagos (15/61), Enugu (10/60) and Kano (9/57). ITPS was distributed to households in urban Lagos (15/60), urban and rural Enugu (10/60 and 15/60, respectively) and urban and rural Kano (9/57 and 15/57, respectively). The majority of houses had plastered (116/178) or mud walls (55/178). All materials were installed by a hired contractor and attached to house walls using nails.

### Household acceptability and lining durability

In Angola knowledge of malaria was assessed among the heads of all 80 households (DL and IRS allocations), using a questionnaire (conducted in Portuguese or Umbundu), prior to installation of the wall materials. At two weeks and one year following wall treatment, the heads of households were interviewed using a knowledge, attitude and practice (KAP) questionnaire. This survey contained questions relating to ease of wall installation, condition of wall and ceiling products, material and colour preference, problems with the materials, whether households would be interested in purchasing the materials (and, if so, at what cost), vector control product preferences and suggested improvements for the materials and installation procedure. In addition, at both follow-ups, interviewers surveyed the houses to evaluate the condition of the wall linings.

**Figure 3:**
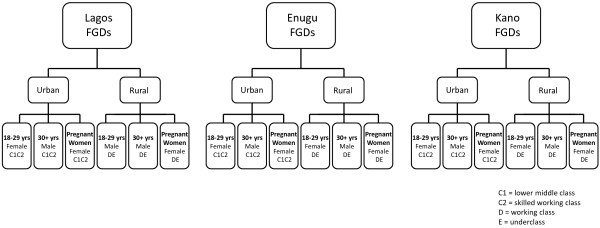
Summary of focus group discussions conducted in rural and urban areas of Nigeria.

In Nigeria before wall installation, a baseline study comprising 18 focus group discussions (FGDs) and 10 in-depth interviews (IDIs) was undertaken to provide insights into consumers’ general knowledge of malaria control, perceptions of wall decorations and attitudes towards the concept of using a durable wall lining for disease control. The FGDs were held in small groups, led by a facilitator, during which members were able to speak freely and spontaneously and did not receive leading phrases to guide their responses. The FGDs were conducted with individuals all belonging to the same sex and age demographic. In each rural and urban area, three separate FGDs were held with 18–29 year old adults (males or females), adults of 30 years and older (males) and pregnant women of any age. Participants for each FGD were recruited based upon their social class, which was categorized according to the National Readership Survey (NRS) social grades [23] (Figure 3).

The IDIs were conducted with individual medical doctors, non-government organization employees (NGOs) and Ministry of Health personnel and covered similar topics to those discussed in the FGDs. Both the FGDs and IDIs were moderated using a structured interview guide and all conversations were digitally recorded. Midway through discussions, the three prototype lining materials were revealed to allow respondents time to critically assess them. After baseline opinions were collected, the heads of each household who received wall linings were interviewed after two weeks and one year, using a KAP questionnaire (based upon the questions used in Angola). Furthermore, at both follow-ups, interviewers surveyed the house to evaluate the condition of the wall installations.

### Data management and analysis

Digitally recorded FGDs and IDIs were transcribed verbatim and field observations were also recorded. Qualitative analysis was conducted according to grounded theory, which allows recurring theoretical generalizations to emerge from the data, as opposed to evaluating the data using a pre-determined hypothesis [24]. Briefly, transcribed passages were initially categorized according to general topics, with additional sub-themes emerging upon further re-reading. Once relationships between themes were established, the text passages were systematically cross-referenced using key words in order to determine the frequency of such associations throughout the transcribed material. Quantitative data derived from KAP questionnaires were summarized using proportions and means.

### Informed consent and ethical considerations

Participants from all study areas were recruited after meeting with and obtaining written consent from the local administrators and community leaders. Written informed consent was also sought from all individuals involved in the FGDs and IDIs and from the heads of households who participated in the trial. All consent forms were reproduced in the local *lingua franca* (Portuguese or Umbundu in Angola and English in Nigeria) to ensure volunteers understood the forms and all aspects of the study were explained. Participants were informed that involvement in the study was completely voluntary and that they could withdraw from the trial at any time without penalty. In addition, at the time of study recruitment, leaflets detailing the symptoms of malaria and recommended precautions (including other forms of vector control) were distributed. This study received approval from the Ethics Committee at the London School of Hygiene and Tropical Medicine.

## Results

### Study participants

In Angola, 61 of the 80 study participants were born in Huambo province and the majority were women (57/80). In Nigeria the average number of individuals in each FGD was ten. At least one member from each of the 178 households that received wall lining installations participated in the FGDs. The IDIs were conducted with seven medical doctors (three from Lagos and two from both Enugu and Kano) and three NGO/Ministry of Health personnel (one from Enugu and two from Kano).

### Knowledge of malaria

Before wall treatment, in Angola general knowledge of malaria among the study participants was low. Less than half of participants (33/80) were aware that malaria and *‘paludismo’* (Portuguese translation of malaria) were the same disease and this figure was lower for women (20/57) than for men (13/23). Malaria was generally considered more serious and a consequence of *‘paludismo’*. However, 81% (65/80) of individuals associated the wet season with an increase in mosquito numbers, 73% (58/80) recognized mosquitoes as the cause of malaria/*‘paludismo’* and 70% (56/80) identified fever as a symptom of malaria/*‘paludismo’*. Most individuals received their information about malaria/*‘paludismo’* from the radio or health posts. By contrast, in Nigeria the level of malaria awareness was very high among rural and urban FGD participants and it was perceived as an inevitable aspect of daily life (Table 1 for supporting quotations).

**Table 1:** Summary of perceived causes of malaria among urban and rural participants in Angola and Nigeria

**Perceived causes of malaria**	**Angola**	**Nigeria**	
	**Urban**	**Urban**	**Rural**
Mosquitoes	41% knew that malaria and *‘ paludismo’* were the same disease	*‘Malaria fever most especially is caused by mosquito bites. Mosquitoes may bite your child or baby and you can contract malaria so this mosquito issue is something that needs to be addressed.’* Male 30+, urban Lagos.	*‘Mosquitoes are the cause of malaria.’* Pregnant female, rural Enugu.
	73% identified mosquitoes as the cause of malaria	*‘If you look at the statistics by WHO and the Federal Ministry of Health, it will amaze you that millions of children die every year of malaria. There are more children dying of malaria than HIV. It is true that people don’t talk about malaria but it is more serious than HIV.’* IDI (Doctor), urban Kano.	*‘You see there is no way mosquitoes won’t be in mud houses.’* Male 30+, rural Lagos.
	70% named fever as a malaria symptom		
Environment	18% identified trash and dirty areas as possible causes of malaria	*‘…and then other causes are uncleanliness, dirty environment, bushy environment and dirty water.’* Pregnant female, urban Lagos.	*‘It is caused by dirt in the environment; mosquitoes feast on this dirt and then inject it into peoples’ bodies, which causes malaria fever.’* Male 30+, rural Lagos.
		*‘I think basically an average Nigerian should have malaria parasites because of our environment.’* Female 18–29 years, urban Lagos.	
Other	10% believed malaria could be transmitted by contaminated food/drink	*‘Stress can bring malaria out faster.’* Female 18–29 years, urban Kano.	*‘Too much sun can also cause malaria.’* Pregnant female, rural Enugu.
	8% implicated water as a source of malaria		

In both study areas, the environment was also considered a major risk factor for malaria transmission. In Angola, after mosquitoes (58/80), trash and dirty areas were identified as possible causes of malaria/*‘paludismo’* by 18% of individuals (14/80). In Nigeria, environmental pollution was implicated as a continuous source of malaria. Misconstrued causes of malaria, including sun, stress, food and water, were reported by a minority of respondents in both countries (Table 1).

### Prevention of malaria

During the pre-wall treatment survey in Angola, participants were asked what precautions they could take to prevent mosquito bites and malaria/*‘paludismo’*. While 48% (38/80) identified insecticide-treated nets (ITNs) as a method of protection from mosquito bites, just 34% (27/80) recognized that ITNs could prevent malaria. Nineteen (of 80) respondents owned a mosquito net, and of these, only 10 nets were treated with insecticide and none within the previous six months. Of the individuals who mentioned mosquito nets as a malaria control method, 26% (7/27) actually owned one. In addition, only one quarter of pregnant women and 17% (14/81) of children under five years old reported sleeping under a bed net at night. Mosquito nets were purchased from the market or at a health post. Secondary malaria control practices were also described, which included house cleaning, taking anti-malarial medication, using insecticide sprays, burning fires/smoke, purifying drinking water and closing doors/windows.

In Nigeria, a range of conscientious and often individualistic prevention methods were described in the FGDs, reflecting the high level of malaria awareness (Table 2). Residents in both rural and urban areas undertook a number of practices, many of which were incorporated into their daily routine. In urban areas, conventional vector control methods, including ITNs, house screening, environmental management and insecticide spraying, were all commonplace. By contrast, rural households described more unorthodox and potentially dangerous practices such as house spraying with kerosene and local dichlorvos pesticide concoctions (‘*ota-piapia’*), using UV lamps and burning pineapple/orange rinds and ant-hills. Choice of vector control method was primarily determined by product affordability. For example, only rural inhabitants reported using mosquito coils. However, both urban and rural participants disliked mosquito coils, believing that they caused cough and catarrh (inflammation of mucous membranes), which were usually associated with the *harmattan* season in Nigeria (West African trade winds). Fumigation using insecticide was considered more costly and only reported in urban households and hospitals. ITNs emerged as one of the most preferred malaria control products, although movement constraints and their limited protection for individuals not sleeping underneath them were identified as potential shortcomings.

**Table 2:** Summary of attitudes towards malaria prevention among urban and rural participants in Angola and Nigeria

**Methods of malaria prevention**	**Angola**	**Nigeria**	
	**Urban**	**Urban**	**Rural**
Insecticide-Treated Nets	48% identified ITNs as a method of preventing mosquito bites	*‘Mosquito nets are a proper preventive measure against mosquitoes*.*’* Female 18–29 years, urban Kano.	N/A
	34% identified ITNs as a method of preventing malaria	*‘For me I don’t like insecticide-treated nets, reason being that you don’t have adequate air flow.’* IDI (Doctor), urban Lagos.	
Environmental Management	23% reported house cleaning as a method of preventing malaria	*‘…the only paramount way to prevent it is mostly through environmental cleanliness. Our environment should be clean so that there would not be all these insects around us.’* Male 30+, urban Enugu.	*‘…the primary answer is to make sure you live in a very hygienic home, make sure you don’t allow stagnant water to stay within your dwelling.’* Male 30+, rural Lagos.
	5% reported purifying drinking water to prevent malaria		
House Screening	3% of households reported closing doors and windows to prevent malaria	*‘The number one thing we need to do to stop their crusade is to net houses and we need to check the number of times we go in and out of the house.’* Male 30+, urban Lagos.	*‘One should use a broom to scare them out of the house and then close the windows so that they won’t be able to come in.’* Male 30+, rural Lagos.
Mosquito Coils/Insecticide Spraying	8% reported spraying insecticide to prevent malaria	*‘…first time I noticed I can’t breathe very well. The second day, I put the mosquito coil on again. I nearly collapsed when I woke up, the thing had choked my heart.’* Female 18–29 years, urban Enugu.	*‘…even it [mosquito coil] is dangerous to children.’* Male 30+, rural Lagos.
		*‘I spray some insecticide because it kills mosquitoes.’* IDI (Doctor), urban Enugu.	
		*‘I dislike some insecticides like Rainbow because of its severe odour, it is pungent and can cause upper respiratory infection.’* IDI (Doctor), urban Kano.	
		*‘It is the mosquito repellent, it tends to make my skin stain and after sometime when you sleep, mosquitoes will still come to bite you.’* IDI (Doctor), urban Lagos.	
Other	23% described taking anti-malarial medications	*‘But what I do when I was on shift was to take fansidar.’* IDI (Doctor), urban Kano.	*‘We always use herbs to prevent malaria.’* Male 18–29 years, rural Lagos.
	5% reported using fire/smoke to prevent malaria	*‘I will put this orange rind in a charcoal pot and burn it and put it in the room and take my children outside and close the window for a short time. I will take it out and bring my children in.’* Female 18–29 years, urban Kano.	*‘The only thing that can drive them away is the fan. If there is light and the fan is on.’* Male 30+, rural Lagos.
		*‘… like the UV light that you plug in and when any mosquito comes in, they are attracted towards the light and they burn.’* IDI (MoH), urban Lagos.	*‘Water and kerosene kills mosquitoes more than any other thing.’* Pregnant female, rural Lagos.

### Perceptions of wall decoration and wall lining concept

**Figure 4:**
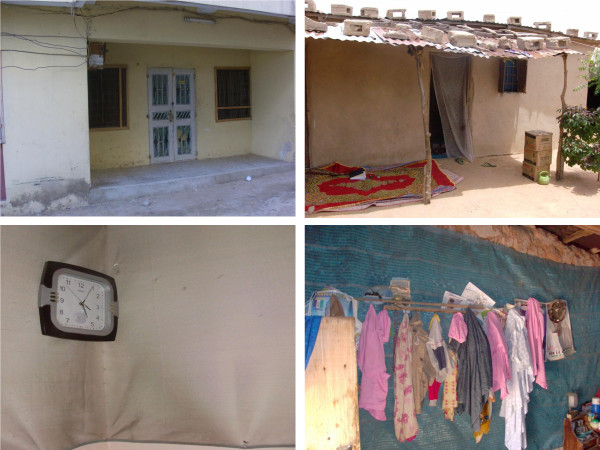
**Differences between urban (left) and rural (right) house exteriors (top) and interiors (bottom) in Kano, Nigeria.** Urban and rural populations in Nigeria disagreed about how a wall should be decorated to be considered attractive. In urban areas (left) wall decorations were minimal. By contrast, in rural houses (right) occupants took pleasure from decorating their walls.

During the FGDs in Nigeria, participants were asked to describe their current wall decorations and to discuss the concept of covering their walls with a lining material. Ultimately, a dichotomy between rural and urban respondents emerged regarding how a wall should be decorated in order to be considered ‘attractive’ (Table 3). In urban areas the use of expensive wall paints, such as gloss and textured paints, appeared prominent. In these settings, wall decorations were minimal and walls were adorned with very few items, limited to calendars, paintings, wall clocks, flowers and family pictures (Figure 4). Inhabitants disapproved of too many decorative items, believing they rendered the wall ‘clumsy’ and ‘unattractive’. By contrast, in rural areas respondents derived pleasure from decorating their walls. Some wall designs represented shrines/memorials to the family, including wedding and graduation photos, etc. Other items commonly hung on the wall included flowers, posters, calendars, wall clocks, paintings, hand-made decorations, radio loud speakers, plates and dishes, clothes and traditional medicines. Rural house occupants used either nails or gum to fix wall decorations. Preference was given to nails because they were more durable, appeared neater and rarely left marks on the walls.

**Table 3:** Perceptions of wall decorations and willingness to pay for durable wall lining materials in Angola and Nigeria

**Perceptions**	**Angola**	**Nigeria**	
	**Urban**	**Urban**	**Rural**
Wall Decoration and Wall Lining Concept	N/A	*‘I don’t like my walls to be congested with things. I only like simple art work.’* Male 30+, urban Lagos.	*‘If you come into my house now, the first thing you will see is the picture of my dad and mum when they wedded*.’ Female 18–29 years, rural Enugu.
		*‘As for me I just love plain walls not because of any religious belief. I just like the natural wall painted.’* Female 18–29 years, urban Kano.	*‘[durable wall lining]…I will be happy having something like that.’* Female 18–29 year, rural Kano.
		*‘I think there is something interesting about the concept that says, it’s going to be active for three to five years. That is something that has not been achieved in Nigeria. I don’t know about other countries.’* Male 30+, urban Kano.	
		*‘In fact it is very fine because of the three to five years duration.’* Female 18–29 years, urban Lagos.	
		*‘If it is not toxic and it is safe especially for children so that I can put it in any part of the house.’* IDI (Doctor), urban Enugu.	
Delivery Systems and Control Product Costs	Before wall installations: majority of households willing to pay over 400KZ for a product to prevent malaria	*‘If the producer can have a distributor or certified people that are selling it but if they put it in the open market, it will not be good, people may be cheated because there are a lot of untreated nets in the market and they can present it and say it is treated and you buying it will not know.’* IDI (Doctor), urban Kano.	*‘It should be in the open markets where everybody can easily get it.’* Female 18–29 years, rural Enugu.
			*‘If it is sold for N2000, People will buy it.’* Pregnant female, rural Lagos.
	Two weeks after wall installations (DL/ITPS households): 37% willing to pay 201-500KZ, 25% willing to pay 501-1000Kz and 24% willing to pay over 1000Kz for DL/ITPS	*‘For my own room, I don’t think I can pay more than N1500.’* Male 30+, urban Lagos.	
			*‘N5000 is okay.’* Male 18–29 years, rural Lagos.
	One year after wall installations (DL/ITPS households): 38% willing to pay 501-1000Kz for DL/ITPS		

Overall, respondents in Nigeria were positive about an alternative approach towards malaria control. Individuals appreciated the concept of a durable wall covering because it would eliminate the need for a daily precautionary routine and it represented a single preventive method. Other aspects, which appealed to participants, were the lining materials’ aesthetics, their potential to eliminate nuisance insects and their long-lasting technology. Concerns that were raised included the safety of children around the materials, potential adverse effects and whether the linings might increase indoor room temperatures.

### Household installation of wall lining materials

In Angola, 50% (30/60) of participants reported no problems installing the materials with the assistance of trained personnel. Twenty-three individuals experienced facial irritation after working with the materials. Four participants described difficulties attaching the ITPS to the ceilings, especially in houses with high ceilings. Four other households reported problems fixing the materials in houses with hard wall surfaces. At two weeks post-installation, the DL and ITPS were still in place in 95% (57/60) of houses (of the three where they were not, two houses had experienced nail failures and one had changed occupancy). Ninety per cent (53/59) of households reported no difficulties with their materials once they were installed. Four individuals, all from houses with low roofs, perceived a rise in indoor room temperature during the day and two participants complained that nuisance rodents ran across the top of the ITPS ceiling.

In Nigeria, the three prototype wall linings were installed by a trained contractor. However, the carpenter was unable to install the lining materials unassisted in 65% (116/178) of households. Additional help was required to hold the lining in place when cutting it to fit the house dimensions and when fixing it to the walls. Other issues encountered by over half of households (100/178) were the need to move/rearrange household furniture during installation and difficulties fixing nails in houses with hard wall surfaces (30/178). The carpenter and house occupants all agreed that the easiest lining to install was the DL, while the most challenging material was the ITPS. Average installation time per house was twice as long in urban (64 minutes) than rural (32 minutes) households.

### Household acceptability of wall lining materials

In Angola, at two weeks after installation, 98% (58/59) of households expressed satisfaction with the materials. The remaining respondent was concerned about the materials’ flammability but did not remove either the wall or ceiling linings. Seventy-three per cent (43/59) of households identified the killing of insects (and a few rodents) as the principal benefit of the DL and ITPS. Thirty-six per cent (21/59) of respondents reported that their house was more attractive because of the materials. Three individuals believed the ITPS ceiling material would provide a shelter during the rainy season. Importantly, the majority of participants (53/59) reported an observable reduction in the number of mosquitoes and other insects in their houses.

One year after installation 27/59 of the DL/ITPS households were lost to follow-up because the Angolan study population was highly mobile; many displaced individuals had settled temporarily in this area as refugees. Of the 32 remaining DL/ITPS households, seven still had their DL and ITPS correctly installed, two other households had only their DL properly installed and 23 no longer had any of the materials installed. Of the 25 households that did not have both correctly installed materials, the majority (22/25) had taken down their DL/ITPS after seven to 12 months. Many participants reported having stored, sold or given away parts of their linings, and the residual scraps of materials were now used to cover peri-domestic areas such as toilets, plants and chicken coops. The most common reasons for removing the linings were that householders felt the insecticide was exhausted and the materials were no longer effective, rodents made noise running on the ceilings and walls, the ITPS ceiling had trapped heat indoors and the materials were dirty and/or had fallen down.

Despite the reduced rate of user compliance after one year, all 32 remaining households still asserted that they had liked having the DL and ITPS materials in their homes. The two most common advantages highlighted by participants were that the DL and ITPS reduced the number of mosquitoes and insects and significantly improved their household aesthetics. All 32 households reported observing a decrease in the number of mosquitoes and nuisance insects in their homes after receiving the wall installations. Interestingly, 21 households could identify a time-point during the year when the materials had stopped noticeably killing mosquitoes. The majority of these households (14/21) believed the DL/ITPS were ineffective after seven to 12 months.

Participants in Nigeria were interviewed after two weeks about the installation and household acceptability of the three lining materials (Table 4). In general, many respondents found all linings rather strange on initial impression, believing that they made their house resemble a studio or cinema hall. Others remarked that the materials narrowed their rooms. In rural areas, where DL was assessed against ITPS, this optical effect was readily overlooked particularly once the efficacy of the lining materials was observed. Rural respondents easily adjusted to the presence of the linings believing they improved their rooms’ aesthetics. However, in urban areas, where all three materials were evaluated, the level of product acceptability was lower, although a number of households did attest to the effectiveness of all three linings. One major advantage over currently available vector control products (ITNs and IRS), identified by many participants, was the lack of smell from all materials.

**Table 4:** Household acceptability of durable wall lining materials two weeks after installation in Angola and Nigeria

**Perceptions**	**Angola**	**Nigeria**	
	**Urban**	**Urban**	**Rural**
Acceptability of wall lining materials	98% of participants expressed satisfaction with the DL/ITPS	*‘It makes the room look like a studio room.’* Male 30+, urban Lagos.	*‘Since we have put that thing, it has beautified my house.’* Female 18–29 years, rural Enugu.
	73% described the DL/ITPS killing insects and rats	*‘For the odour, I think I am okay with it because it is not bad.’* Male 30+, urban Lagos.	*‘The products also killed lizards because there was a lizard in my room which I was not aware of until I saw it moving on the wall and as soon as it touched the product it couldn’t move again and it was killed.’* Male 30+, rural Lagos.
	90% reported a reduction in the number of mosquitoes and other insects in their houses	*‘I have been closing my door because if people had seen it they would be asking me questions.’* Pregnant female, urban Lagos.	*‘The thing is picking insects the way I can’t explain. It’s picking them like a magnet. It was very very effective.’* Female 18–29 years, rural Enugu.
	36% believed their house was more attractive because of the DL/ITPS	*‘In fact the first day they brought it when my husband came back, he said that I have disfigured the wall.’* Female 18–29 years, urban Lagos.	*‘We like everything about this product.’* Male 30+, rural Lagos.
		*‘The mosquito is not that bad that I should now disfigure my wall.’* Pregnant female, urban Enugu.	
Installation of materials	50% experienced no problems installing the materials with the help of trained staff	*‘You might not be able to do it on your own, an expert has to do it for you.’* Female 18–29 years, urban Lagos.	*‘*… *It [nails] spoils the wall’.* Female 18–29 years, rural Enugu.
	90% reported no problems with their materials once they were installed	*‘Whenever you use nails, you destroy the beauty or the particular face of that wall.’* Male 30+, urban Lagos.	
Suggested improvements	Respondents suggested using the DL on the ceilings instead of the ITPS	*‘Put designs like flowers to beautify it.’* Female 18-29 years, urban Kano.	*‘We should have bought a tyroid and nailed it to the wall before fixing the product.’* Male 30+, rural Lagos.
		*‘It can even come in a form of painting.’* Male 30+, urban Lagos.	
		*‘It will be like a long projector. In the afternoon, nobody notices anything was there, when the evening time comes, you pull it down.’* Male 30+, urban Lagos.	*‘The gum cannot stay on the walls, it will fall down. I think nails are better.’* Male 30+, rural Lagos.
		*‘If the lining can be done well in such a way that it is accurately measured and cut well and stretched before it is ready for sale because you find out that when you are fixing it to the wall if you don’t stretch it properly there will be some space it will not cover.’* Female 18–29 years, urban Kano.	

Ultimately, the two principal arguments raised by urban households against the use of wall linings were installation feasibility and aesthetic value. Respondents believed the materials disfigured the houses, rather than beautified them. They also felt that the materials were too plain to be used as wall coverings and suggested that they should have patterns/pictures or be produced as wall paint in different colours. Generally, urban populations repaint their houses every one to two years; thus the DL was perceived to spoil the walls. Concerns were also raised by urban households about the number of nails required for installation and the obligation to cut the linings to fit house dimensions. Respondents expressed strong objections to the use of too many nails, as they may erode parts of the wall and require future renovations. Suggestions given to improve installation included the use of ‘tyroids/battons’ (small wooden planks) to secure the lining edges or ‘rollers’ that would enable the product to move up and down as desired by the user, and to manufacture the linings in different pre-determined room sizes.

In rural areas, the use of nails was more acceptable, but concerns were raised about their size. For old houses, long nails may cause greater damage to the walls and so the use of one to two inch nails was suggested. Regarding the ability to install the product, most respondents in the urban areas stated that they would need professional help and were unable to fix the product unassisted. By contrast, rural participants declared that they would fix the material by themselves with help from their friends. In these areas, individuals are responsible for minor house renovations, such as repairing leaking roofs, broken chairs/benches, etc., usually to save costs incurred by paying a carpenter.

One year after installation, 87/178 households were lost to follow-up; similar numbers of households remained across all three study sites (28/91 in Lagos, 25/91 in Enugu and 38/91 in Kano). In Lagos and Kano the majority of remaining households were in rural areas (20/28 and 24/38, respectively) while in Enugu more urban houses (14/25) were available for follow-up. Most of the remaining 91 households had received DL (n=55/91; 22/28 in Lagos, 16/25 in Enugu and 17/38 in Kano) followed by wall netting (n=21/91; 6/28 in Lagos, 6/25 in Enugu and 9/38 in Kano) and finally ITPS (n=15/91; 3/25 in Enugu and 12/38 in Kano). After one year of field use, 67% (n=61/91; 16/61 urban and 45/61 rural) of remaining households still had their wall linings installed. Of the 15 remaining households that had received ITPS, all houses still had their materials correctly installed. By contrast 35% (19/55) and 52% (11/21) of remaining DL and wall netting households, respectively, had removed their wall linings.

Ninety-five per cent (58/61) of households with their linings still installed claimed that the materials’ effectiveness against insect vectors had declined over the year. The majority of households identified the killing of mosquitoes (79%) and nuisance insects (51%) and the improvement of house aesthetics (28%) as the primary benefits of the wall linings. Of those households that had removed their linings during the year (30/91), 37% (n=11/30; 3/11 wall netting houses and 8/19 DL houses) removed the material to renovate their houses and 23% (n=7/30: 3/11 wall netting houses and 4/19 DL houses) did so because they no longer considered the wall lining effective. Ninety per cent (55/61) of those who still had installed linings and 60% (18/30) of those who had removed their materials claimed that they would like to have another lining installed in the future, if offered. Of those who did not want to re-install another wall lining (24/91), 42% maintained that it did not add decorative value to the house, while 34% believed it had spoiled the room and damaged the walls.

### Choice of vector control product

In Nigeria, FGD and IDI participants were asked to comment on the attractiveness of the three prototype durable wall linings (DL, wall netting and ITPS) (Table 5). In all discussions, the DL emerged as the most desirable of the three lining materials. Respondents appreciated its aesthetics because it closely resembled a traditional mat (‘*Hausa*’) commonly used in Nigerian homes. However, this feature also raised concerns that the product could be easily forged. Some participants were worried that it was too fragile and children could damage it. In addition, urban respondents stated that the weaving made it look more suitable for rural people.

**Table 5:** Summary of favourable and unfavourable characteristics of durable wall lining materials identified during focus group discussions and in-depth interviews in Nigeria

**Durable Wall Lining Material**	**Overall Impression**	**Favourable Characteristics**	**Unfavourable Characteristics**	**Nigeria**	
				**Urban**	**Rural**
Shade Cloth DL	Resembles traditional Nigerian house mat (*‘Hausa’*)	It looks patterned	It will be easy to imitate	*‘When you get to my house now, my children have torn the one at home.’* Female 18–29 years, urban Kano.	*‘It looks like a Hausa mat.’* Male 18–29 years, rural Lagos.
	Also resembles traditional Yoruba clothes (*‘Aso oke’*)	Large mesh size increases air ventilation	Children can easily damage it	*‘Personally I have no comment on this one because definitely I can’t use this one. It is meant for the mud houses.’* IDI (Doctor), urban Lagos.	
	Is more suitable for rural households				
ITPS	Resembles cement packing sack (‘bagco super sack’)	Could be mistaken for wall paper, if closely attached to the wall	It is too plain	*‘Yes, it looks more like wall paper.’* Female 18–29 years, urban Kano.	*‘It looks like a bagco bag.’* Male 30+, rural Lagos.
	Is also similar to sacks used for spreading grain	It is well knitted	It is too thick and may trap heat indoors	*‘It is too plain, pictures or artwork or painting should come on it.’* IDI (Doctor), urban Lagos.	*‘This one does not have any space therefore it will cause more heat.’* Male 30+, rural Lagos.
				*‘The sacky nature, it is too thick.’* Female 18–29 years, urban Kano.	
Wall Netting	Resembles common window nets	It is manufactured in different colours	It may not attach easily to the wall	*‘Different colours so that you can choose the one you want.’* Female 18–29 years, urban Enugu.	*‘The other one doesn’t have spaces, this one has spaces.’* Male 18–29 years, rural Lagos.
		Large mesh size increases air ventilation	It is considered unusual to hang nets on walls and may invite unwanted attention	*‘Because the net is not supposed to be on the wall.’* Male 30+, urban Lagos.	*‘Having it on my wall is impossible. I won’t like it.’* Pregnant female, rural Lagos.
		Light-weight fabric appears transparent	It will be difficult to distinguish the insecticide-treated wall netting from the already existing untreated window nets	*‘This looks like a mosquito net, this is fine but is this for the wall too?’* IDI (Doctor), urban Kano.	
				*‘Ventilation matters a lot.’* Male 30+, urban Lagos.	

ITPS was well received because of its similarity to wallpaper. However, it was associated with a traditional sack (‘bagco super sack’) used for packing cement, which had negative connotations in Nigeria. It was also condemned for its thickness, which could potentially trap heat in the room.

Wall netting emerged as the least popular choice because of doubts regarding whether it was actually treated with insecticide. Some respondents stated that they would never put it on the wall as such materials are meant for windows and doors only. However, wall netting was commended for being available in different colours that could match the room colour and for having holes/pores on the surface to allow air passage.

These preferences were also apparent at the one year follow-up where householders were asked to rank the three materials according to the following attributes: convenience, ease of installation, aesthetic value, effectiveness against insects, durability, colour/appearance and safety. DL was ranked first for all of these qualities, followed by ITPS and lastly wall netting.

At two weeks after installation in Angola, 97% (57/59) of DL/ITPS households preferred the DL colour they received. Households were randomly allocated blue, green or orange DLs. However, the distribution of colours among the community was disproportionate with most households receiving blue DLs (26/59). Orange and green linings were allocated to 19 and 14 households, respectively. Among the 20 individuals who received IRS, when offered a choice of DL colour, the majority (11/20) reported a preference for blue. When offered the choice between ITNs or DL/ITPS, the majority (56/59) preferred the DL/ITPS and cited the following reasons: the DL/ITPS covered the entire house, killed all the mosquitoes and nuisance insects, improved the house aesthetics and the ITPS on the ceiling provided protection from rain and dust. The three individuals who preferred ITNs felt the protection they would receive from this product would last longer, that the nets would be easier to re-treat with insecticide and, even without insecticide, nets would still function as a physical barrier against mosquitoes. Once the wall lining insecticide reservoir was exhausted, these individuals believed they would no longer be protected against mosquitoes. Fifty-seven (out of 59) of the DL/ITPS recipients preferred the DL/ITPS to IRS. Of the 20 IRS households, 17 preferred the DL/ITPS to IRS and a number of IRS participants requested DL and ITPS from the programme staff.

One year after installation the majority of remaining DL/ITPS households (30/32) and IRS households (17/20) still preferred insecticide-treated materials over IRS. Again, the most common advantages identified by householders were that the DL/ITPS improved household aesthetics and killed nuisance insects and mosquitoes. When the remaining 32 DL/ITPS households were offered a choice between DL/ITPS and ITNs, the majority still preferred DL/ITPS (25/32). The principal advantage of DL/ITPS over ITNs, identified by households, was the protection the DL/ITPS provided for all household members. When asked what features participants would like to change about the materials to improve them, only three respondents replied and all suggested that the DL be used on the ceiling as well as the walls. No one could offer an alternative method of installation stating that they would either buy nails (26/32) or they would not install the material if nails were not provided (6/32).

### Perceptions of delivery systems and control product costs

In Angola, at two weeks after installation, 58 out of 59 DL/ITPS recipients reported a desire to buy the DL and ITPS if they were retailed. The remaining participant who would not buy the materials cited lack of money as the reason. All 20 households that received IRS indicated they would also be interested in purchasing the DL and ITPS materials. In addition, the majority of participants (54/59) stated that their family and friends would like to buy the materials for their houses. At one year post-installation, the majority of participants (28 out of the remaining 32 households) still expressed a desire to buy the DL and ITPS if they were available at market.

Before wall installations, households in Angola were asked how much they would be willing to pay for a product that would prevent malaria. Thirty-two households were unsure, while others were willing to pay between 201-400Kz (US$2.2–4.5) (8/60) or over 400Kz (US$4.5) (10/60). Of the 19 individuals in the community who owned mosquito nets, ten paid between 201-400Kz (US$2.2–4.5) for them, seven individuals received theirs for free and two paid over 400Kz (US$4.5). Following wall installations, the majority of both DL/ITPS (22/59) and IRS (11/20) recipients were willing to pay 201-500Kz (US$2.2–5.6) for the insecticide-treated materials. However, 25% (15/59) of DL/ITPS households were willing to pay 501-1000Kz (US$5.6–11.2), while 24% (14/59) would pay more than 1000Kz (US$11.2) for the DL/ITPS. One year after installation, just over one-third of remaining households (12/32) were willing to pay 501-1000Kz (US$5.6–11.2) for the DL/ITPS.

In Nigeria respondents were primarily concerned with the possibility of forging the wall linings as they closely resembled existing products. Suggestions were made for the materials to come with a manufacturer’s or health authority endorsement, such as a logo, and to be sold in selected and trusted establishments, including pharmaceutical stores, department stores, authorised outlets, etc. However, some participants also commented that if the wall lining materials were sold only in these retailers, rural inhabitants might not buy them as these places are regarded as very expensive. Instead, it was suggested that authorised distributers be present in open markets as well as outlets. Interestingly, when considering pricing, rural respondents were willing to pay more than urban individuals for the wall lining materials (US$12-$40 versus US$4-$24, respectively) (Table 3).

## Discussion

The principal aims of these pilot studies were to identify a highly desirable material to develop into a durable wall lining product and to assess the levels of product acceptability, feasibility of installation and willingness to pay among different socio-economic communities afflicted by endemic malaria.

Initially, households in both study sites were very receptive to the concept of a new long-lasting vector control product. In Nigeria, malaria awareness was very high, as evidenced by the number and variety of disease control methods undertaken by rural and urban households. In these areas malaria prevention was habitual to the extent that occupants would restrict house entry in the evenings to prevent incoming mosquitoes. Wall linings were well received by these communities because they represented a single control method that could potentially alleviate their daily inconvenience. By contrast, in Angola knowledge of malaria was more rudimentary. While most study participants were aware that mosquitoes transmit malaria, only a minority recognized that mosquito nets could protect from malaria. Consequently, ITN coverage was low across the general population and particularly among target groups of pregnant women and children under five. These observations highlight the need for greater investment in education and the promotion and provision of malaria vector control products in Angola. This paucity of basic disease knowledge likely reflects the previous political instability in the region. At the time of this trial, Angola had just emerged from 27 years of successive civil wars, where malaria vector control programmes and operational studies had been interrupted for decades.

A clear division between urban and rural attitudes towards wall linings became apparent following wall installations. In Angola, urban acceptability of DL/ITPS at the two week follow-up was high. However, between seven to 12 months after installation, more than three quarters of households had removed their materials, maintaining that they were no longer effective against insect vectors. These observations suggest that community compliance was principally determined by perceptions of entomological efficacy. Interestingly, this time-point coincided with the end of the rainy season in Angola; therefore these reports of ineffectual wall linings may reflect seasonal fluctuations in mosquito biting. If user acceptability had also been influenced by genuine appreciation of the wall linings’ aesthetics, it is likely that more households would have retained their materials during the dry season for decorative purposes only. It should be noted that the DL and ITPS assessed during this trial were prototypes and no bioassays were undertaken to corroborate perceived entomological efficacy. Parallel community trials in Ghana and Equatorial Guinea of second generation DL demonstrated no significant loss of bioefficacy after one year of field use [15].

In Nigeria, a similar attitude and pattern of behaviour emerged among urban participants. Shortly after installation, the majority of urban households widely acknowledged the efficacy of all three prototype wall lining materials against insect vectors but were eventually unwilling to overlook their objections to the linings’ aesthetics and installation feasibility. For these communities, the perceived threat of malaria was not sufficient to justify ‘defacing’ their walls with a lining material. By contrast, the materials were well received by rural households in Nigeria throughout the study year. These communities commented favourably on the linings’ aesthetic value and efficacy against malaria vectors/nuisance insects. This high level of acceptability was also apparent at the one year follow-up, where the majority of households which still had their wall linings installed were located in rural areas. After one year, as in Angola, some depreciation in entomological efficacy was reported by Nigerian participants who still had correctly installed linings. However, most householders in Nigeria who had removed the wall materials had done so to refurbish their houses and a few because they deemed the material no longer effective.

The behaviour of urban populations from Angola and Nigeria over the study year highlights the importance of perceived entomological efficacy and aesthetic value as key determinants of wall lining acceptability. Ultimately, the wall lining must be desirable enough for households to want to retain it for a number of years on their walls without further encouragement. Experimental hut trials indicate that wall linings act against indoor-resting vector populations in a similar manner to IRS [25-27]. Thus, to significantly impact on malaria transmission, the maintenance of high household coverage with wall linings across entire communities would be crucial for success. The poor user compliance among urban participants suggests that wall linings would not be readily adopted or sustained in towns or cities. The higher level of acceptability among rural inhabitants in Nigeria indicates that these populations are a more appropriate target demographic. The rural communities were more severely affected by malaria, reported undertaking a number of ineffectual daily vector control practices and would benefit the most from measures that reduce disease morbidity and improve quality of life. From an installation perspective, rural populations presented less of a logistical challenge. In the urban Nigerian households, objections were raised against installation feasibility whereas rural communities approached the lining installations with a sense of ‘camaraderie’, as inhabitants regularly assist one another with house repairs.

Regarding choice of lining material, in Nigeria, where three prototype lining materials were evaluated, DL was indisputably more popular than both wall netting and ITPS. It was considered the easiest to install by the carpenter, received the most recommendations at the FGDs/IDIs because of its similarity to traditional Nigerian materials, and was ranked highest by participants at the one year follow-up. The opinions gathered from the FGD/IDIs regarding the materials’ physical characteristics also revealed some interesting observations. The close resemblance of DL and ITPS to locally available materials was unanticipated, as was the perceived embarrassment associated with hanging bed netting on house walls. These results emphasize the importance of early user evaluation to identify any aspects of a product that might adversely affect user acceptability.

In Angola both the DL and ITPS materials received positive feedback from participants. When offered the choice of vector control product (DL/ITPS, ITNs or IRS), the majority of both the DL/ITPS and IRS households chose DL/ITPS at both follow-ups. This preference among the IRS households was evident as a number of participants actively petitioned staff members to also receive DL/ITPS. When questioned about willingness to pay, despite the relatively low importance assigned to vector control before wall installations, the majority of Angolan participants stated that they were prepared to spend up to a fifth of their weekly income (~400Kz) [28] for a product that would prevent malaria. Following wall installations, the amount recipients were willing to pay was similar, while a minority was ready to part with more money (>1000Kz). Considering the poor rate of user compliance among participants, these results may not be a meaningful reflection of willingness to buy such materials in this area. In Nigeria rural participants were prepared to pay more for the wall linings than their urban counterparts and this likely reflects both the importance of malaria control in these communities as well as the aforementioned higher levels of household acceptability. The complexity of designing suitable distribution systems to reach rural communities was highlighted by respondents in the FGDs/IDIs. Retailing a wall lining in open/local markets during product infancy would render it vulnerable to imitation, especially given the close similarity of these materials to locally available ones. Likewise, selling/dispensing such products exclusively through controlled distribution channels could reduce access to potential consumers. Offering a wall lining product at an affordable price will be critical, especially since subsidized ITNs are considered beyond the financial constraints of most rural individuals [29]. It is likely that to achieve high coverage with wall linings a combination of distribution strategies, including social marketing and sensitization through the retail sector, the provision of micro-financed subsidized products from health facilities [30] and mass distribution to the poorest and hardest to reach communities will have to be considered in the future.

### Study limitations

While these preliminary studies are important for early assessment of user preference to improve product design, there are several shortcomings in the reported study, which should be considered when interpreting the results. In both study sites the interviewers were associated with the installation process, making it probable that a proportion of respondents’ answers were not as objective as they might have been had the interviewers been entirely anonymous. In addition, a follow-up study comprising entomological indices, such as indoor and outdoor resting catches of mosquitoes, is needed to substantiate the perceived entomological effects, which were likely influenced by vector population seasonality and intervention coverage level. The opinions expressed by the urban respondents in both study sites are specific to these populations and are not necessarily representative of all urban attitudes towards wall linings and malaria disease control in endemic areas. Wall linings need to be evaluated across a broader geographic range of urban communities before all urban populations are excluded as potential beneficiaries. Importantly, the results of this study also caution the interpretation of household acceptability measurements derived from questionnaires. For example, in Angola, despite very poor household compliance after one year, participants at this time-point still reported positive DL/ITPS feedback. Therefore, it could be argued that a more accurate and sincere estimate of user acceptability is actually household compliance rate. The results of this study have been interpreted with the latter limitation in mind.

## Conclusions

Insecticide-treated durable wall lining is a new vector control product designed to offer dual benefits of protection from vector-borne diseases for all members of a household while also improving interior decoration. User compliance among different urban and rural populations was evaluated and found to be dependent on a variety of factors, particularly perceptions of entomological efficacy and appreciation of the materials’ aesthetics. Household acceptability of the wall linings was polarized between communities, with rural participants emerging as the prominent potential target consumer group. Of the three prototype lining materials assessed (DL, ITPS and wall netting), DL was the most popular because of its ease of installation and resemblance to local materials. If the levels of acceptability among rural households in this study are readily sustainable and reproducible, DL has the potential to become a viable long-lasting community-level alternative to IRS in malaria endemic areas.

## Competing interests

The authors received financial support from The Mentor Initiative and Durable Activated Residual Textiles S.A. (DART S.A.) to conduct the study but have no competing or commercial interests with either company. Neither of these commercial parties played any role in data analysis, interpretation of results, decision to publish or preparation of the final manuscript.

## Authors’ contributions

The field trials were initiated by DART S.A. and The Mentor Initiative and conducted in collaboration with NPM, AOA and TSA. Data was consolidated, interpreted and analysed retrospectively and independently by LAM and MR. LAM and MR wrote the manuscript. NPM, AOA and TSA revised the final manuscript. All authors read and approved the final manuscript.
